# Multimodal therapy for category III chronic prostatitis/chronic pelvic pain syndrome in UPOINTS phenotyped patients

**DOI:** 10.3892/etm.2014.2152

**Published:** 2014-12-19

**Authors:** VITTORIO MAGRI, EMANUELA MARRAS, ANTONELLA RESTELLI, FLORIAN M.E. WAGENLEHNER, GIANPAOLO PERLETTI

**Affiliations:** 1 Urology Secondary Care Clinic, Azienda Ospedaliera Istituti Clinici di Perfezionamento, Milan, Italy; 2 Department of Theoretical and Applied Sciences, Biomedical Research Division, Università degli Studi dell’Insubria, Busto Arsizio/Varese, Italy; 3 Microbiology Unit, Fondazione IRCCS Ospedale Maggiore Policlinico Mangiagalli Regina Elena, Milan, Italy; 4 Department of Urology, Pediatric Urology and Andrology, Justus Liebig-University, Giessen, Germany; 5 Department of Basic Medical Sciences, Faculty of Medicine and Health Sciences, Ghent University, Ghent, Belgium

**Keywords:** prostatitis, chronic bacterial prostatitis, chronic pelvic pain syndrome, Meares and Stamey test, azithromycin, ciprofloxacin, alfuzosin, *Serenoa repens*, NIH-CPSI, UPOINT, UPOINTS, sexual dysfunction

## Abstract

The complex network of etiological factors, signals and tissue responses involved in chronic prostatitis/chronic pelvic pain syndrome (CP/CPPS) cannot be successfully targeted by a single therapeutic agent. Multimodal approaches to the therapy of CP/CPPS have been and are currently being tested, as in the frame of complex diagnostic-therapeutic phenotypic approaches such as the urinary, psychosocial, organ-specific, infection, neurological and muscle tenderness (UPOINTS) system. In this study, the effect of combination therapy on 914 patients diagnosed, phenotyped and treated in a single specialized prostatitis clinic was analyzed. Patients received α-blockers, *Serenoa repens* (*S. repens*) extracts combined or not with supplements (lycopene and selenium) and, in the presence of documented or highly suspected infection, antibacterial agents. Combination treatment induced marked and significant improvements of National Institutes of Health Chronic Prostatitis Symptom Index (NIH-CPSI) prostatitis symptom scores, International Index of Erectile Function (IIEF) sexual dysfunction scores, urinary peak flow rates and bladder voiding efficiency. These improvements, assessed after a 6-month course of therapy, were sustained throughout a follow-up period of 18 months. A clinically appreciable reduction of ≥6 points of the total NIH-CPSI score was achieved in 77.5% of patients subjected to combination therapy for a period of 6 months. When the patients were divided in two cohorts, depending on the diagnosis of CP/CPPS [inflammatory (IIIa) vs. non-inflammatory (IIIb) subtypes], significant improvements of all signs and symptoms of the syndrome were observed in both cohorts at the end of therapy. Intergroup comparison showed that patients affected by the IIIa sub-category of CP/CPPS showed more severe signs and symptoms (NIH-CPSI total, pain and quality of life impact scores, and Qmax) at baseline when compared with IIIb patients. However, the improvement of symptoms after therapy was significantly more pronounced in IIIa patients when compared with IIIb patients. In contrast to current opinion, the evidence emerging from the present investigation suggests that the inflammatory and non-inflammatory sub-categories of CP/CPPS may represent two distinct pathological conditions or, alternatively, two different stages of the same condition. In conclusion, a simple protocol based on α-blockers, *S. repens* extracts and supplements and antibacterial agents, targeting the urinary, organ specific and infection domains of UPOINTS, may induce a clinically appreciable improvement of the signs and symptoms of CP/CPPS in a considerable percentage of patients. In patients not responding sufficiently to such therapy, second-line agents (antidepressants, anxiolytics, muscle relaxants, 5-phosphodiesterase inhibitors and others) may be administered in order to achieve a satisfactory therapeutic response.

## Introduction

Chronic prostatitis/chronic pelvic pain syndrome (CP/CPPS) is a complex condition, characterized by uncertain etiology and by limited response to therapy. CP/CPPS affects men of all ages, and can significantly impair the quality of life (QoL) and the social functioning of patients.

CP/CPPS is characterized by a wide spectrum of symptoms, including pain in the pelvic region, irritative and obstructive voiding symptoms, ejaculatory pain, sexual dysfunction, depression and psycho-social maladjustment amongst others (1).

The failure to individuate a single etiological agent has hampered the identification of curative interventions for CP/CPPS. It has been hypothesized that infection (occult or non-culturable infection included), as well as genetic, anatomical, physiological, neurological and immunological factors may be involved (alone or combined) in the pathogenesis of CP/CPPS. In this regard, experts consider that different cases of CP/CPPS are likely to have different etiological determinants and different disease progression pathways (2). According to Shoskes *et al*, the etiological determinants of CP/CPPS are likely to trigger tissue and cellular responses that include inflammation and the upregulation of cytokine expression and release. Inflammatory injury may damage tissue components such as nerves and blood vessels, in turn causing pain that may produce contraction of pelvic smooth and skeletal muscles, finally leading to lower urinary tract symptoms, ejaculatory pain and pain in other regions, including the lower back and abdomen (3). Prolonged pain may lead to neurogenic inflammation and peripheral and central sensitization.

It is evident that such a complex network of etiological factors, signals and cellular responses cannot be successfully targeted by a single therapeutic agent. Only in very few cases [reviewed in (4)] can a single compound attenuate the symptoms of CP/CPPS, and the failure of single-agent therapy was denounced as early as in the year 2004 by Nickel *et al* (5). As a consequence, research efforts have been focused on the design of new multi-modal therapeutic strategies addressing the wide array of CP/CPPS signs and symptoms (6).

In order to design optimal symptom-directed therapeutic protocols, the clinical phenotype of each CP/CPPS patient should be carefully assessed. A novel algorithm called UPOINT (an acronym standing for the urinary, psychosocial, organ-specific, infection, neurological and muscle tenderness domains involved in the syndrome) has been validated by a number of independent research groups, and is currently being tested in daily clinical practice worldwide in its original form, or modified to include a sexual dysfunction domain (UPOINTS) (7–12).

Following validation of the novel algorithm at the diagnostic level, a pilot prospective study focusing on therapy demonstrated that a high fraction (84%) of patients treated by targeting each positive UPOINT domain had a clinically appreciable improvement of CP/CPPS symptoms (7).

Since the year 2000 our research group has adopted a multimodal approach to treat CP/CPPS. α-adrenergic receptor blockers, antibacterial agents, *Serenoa repens* extracts and various supplements active on the prostate gland have been administered to a large number of patients, whose follow-up data have been recorded in a database of ~1,600 men affected by different forms of prostatitis. The present study was aimed at retrospectively evaluating the long-term effect of combination therapy on CP/CPPS patients, and to attempt a comparison with other studies based on UPOINT-driven therapy.

## Patients and methods

The present study was performed on patients who were subjected to diagnostic and therapeutic protocols routinely adopted in our clinical practice (8). Patients provided written informed consent to anonymous publication of their clinical data. According to the Italian regulations (Determinazione AIFA 20/3/2008, GU 76), the protocol describing the present observational study was notified to the Ethical Committee of the Principal Investigator’s hospital (authorization 26/10/2009, ICP register: 244).

### Diagnostic procedures

The clinical data of 914 fully compliant patients, diagnosed in a single urology outpatient center specialized in treatment of prostatitis syndromes, and meeting a number of selective inclusion criteria were retrospectively analyzed.

Patients between 20–59 years were included in this study if they exhibited at a first visit signs and symptoms of category III CP/CPPS, according to National Institutes of Health (NIH) criteria (NIDDK Chronic Prostatitis Workshop, 1995).

History collection, clinical, microscopic, microbiological and instrumental diagnosis of patients, urological visits as well as inclusion/exclusion criteria have been described in detail in a previous report of the present study (8), focusing on the diagnosis and UPOINTS phenotyping of CP/CPPS patients. Urinary peak flow rate (Qmax) and the percentage bladder voided volume (%BVV) were assessed in each patient as previously described (8).

The severity of the chronic prostatitis symptoms was scored by means of an Italian validated version of the NIH Chronic Prostatitis Symptom Index (NIH-CPSI), addressing pain and voiding symptoms, and the impact of the disease on patients’ QoL (13). A reduction of ≥6 points of the total NIH-CPSI score was considered as a clinically appreciable improvement of CP/CPPS symptoms (14). All CP/CPPS patients were phenotyped according to the UPOINTS system, as previously described (8).

The International Index of Erectile Function (IIEF) questionnaire was adopted to assess the erectile function of patients (15). Mild to severe erectile dysfunction (ED) was defined as a sum of the scores for IIEF questions 1–5 and 15, which in total were inferior to 26 (15).

### Study design

At time-point V0 (visit zero), after complete clinical and microbiological assessments, patients received a full course of combination pharmacological therapy. Microbiological eradication of pathogens was assessed at the end of a 4-week cycle of antibacterial therapy. All other tests were performed after 6 months of continuous combination therapy: at time-point V6 (visit 6 months), patients were subjected to a complete diagnostic protocol, including microbiological and clinical evaluations. Follow-up visits, including instrumental assessments, questionnaires and urological visits, were performed 12 months (time-point V12) and 18 months (time-point V18) after the start of therapy.

### Pharmacological treatment

Starting from time-point V0, patients were treated for 6 months with a combination of drugs, already tested in a variety of other settings (16).

Combination therapy included a daily dose of the α-adrenoceptor blocker alfuzosin (10 mg, extended-release formulation; various brands chosen by the patient or general practitioner) and a *S. repens* extract [640 mg/day; from patient choice of Permixon^®^ (Pierre-Fabre Pharma, Milan, Italy), SABA^®^ (Lampugnani Farmaceutici, Milan, Italy) or Serpens^®^ (Laboratorio Italiano Biochimico Farmaceutico Lisapharma, Como, Italy). The latter was administered alone, or in the form of a combined tablet preparation (Profluss^®^; Konpharma, Rome, Italy) including *S. repens* (640 mg/day), lycopene (10 mg/day) and selenium (100 μg/day) (17,18).

The patients for which positive microbiological cultures of prostate-specific specimens (expressed prostatic secretions and/or post-massage voided urine) were obtained (positive UPOINTS infection domain) received in addition an oral antibacterial therapy with a fluoroquinolone (ciprofloxacin 750 mg/day) and a macrolide (azithromycin 500 mg/day, the first 3 consecutive days of each treatment week) for 4 weeks (16).

### Statistical analysis of data

Due to the size of the patient population, and since the distribution of the NIH-CPSI scores and uroflowmetry data in the patient population was normal (not shown), the normality assumption was applied to all datasets. When ordinal scales were analyzed, both mean and median scores were calculated as measures of the central tendency of the patient populations, and standard deviations and interquartile ranges are shown as measures of data dispersion.

Intergroup differences were calculated by the Mann-Whitney test (questionnaire scales) or the t-test for unpaired heteroscedastic samples (continuous variables). Intragroup differences were analyzed by a paired t-test (continuous variables) or by the Wilcoxon signed-rank test (ordinal scales).

When two treatment strategies were compared, the analysis of covariance (two-way ANCOVA) was applied to analyze inter-arm differences. This test was also applied to comparisons of questionnaire scores showing non-skewed data distributions, according to Vickers (19). Differences in patient proportions at specific study time-points were analyzed by a two-tailed Z test.

The VassarStats on-line statistics platform (http://vassarstats.net) and the VassarStats ANCOVA Excel spreadsheet (http://vassarstats.net/exl/ancovaM.xls) were used for analysis of data. P<0.05 was considered to indicate a statistically significant result.

## Results

### Total patient population

A population of 914 patients, meeting the inclusion criteria for the present study, was extracted from our clinical database. Patients were affected by category III CP/CPPS, of the inflammatory (type IIIa, n=367) or non-inflammatory (type IIIb, n=547) sub-categories (20).

Patients were phenotyped according to the UPOINTS system (8). Similarly to a preliminary report of the present study, and to other international trials (2,8), 57.60, 33.77, 97.05, 9.94, 46.23, 68.52 and 49.07% of patients exhibited a positive urinary, psychosocial, organ-specific, infection, neurological, muscle-tenderness and sexual domain, respectively.

Table I summarizes the clinical findings of the total patient population at enrollment (V0), at the end of a 6-month cycle of therapy (V6), and 6 or 12 months after the end of therapy (time-points V12 and V18, respectively).

Total NIH-CPSI scores decreased significantly from a baseline mean value of 20.91 to 9.87 at time-point V6 and to 8.15 and 7.62 at time-points V12 and V18, respectively (P<0.0001 for all paired comparisons vs. V0, Wilcoxon signed rank test). The difference between values at V6 and at V12 or V18 was also significant (P<0.0001, Wilcoxon).

A clinically appreciable reduction of ≥6 points of the NIH-CPSI score (14) was assessed at the end of therapy in 77.5% of patients (n=708).

A 57% reduction of NIH-CPSI pain scores was calculated in this population at the end of therapy. Further significant pain reductions were observed at time-points V12 or V18 (Table I).

**Figure 1:**
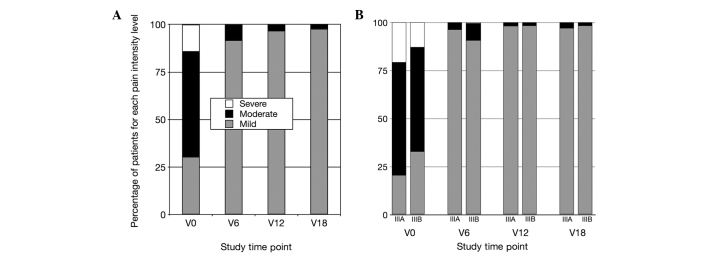
Distribution of pain intensity scores, assessed in CP/CPPS patients with the NIH-CPSI. NIH-CPSI sub-scores were assigned to three increasing pain levels, according to Wagenlehner *et al* (21) as follows: Mild pain, 0–7 points; moderate pain, 8–13 points; severe pain, 14–21 points. (A) Pain intensity distribution in the total patient population. (B) Pain intensity in patients affected by the inflammatory (IIIa) or non-inflammatory (IIIb) sub-categories of CP/CPPS. Data are shown at enrollment (V0), at the end of therapy (V6), and at follow-up time-points (V12, V18). CP/CPPS, chronic prostatitis/chronic pelvic pain syndrome; NIH-CPSI, National Institutes of Health Chronic Prostatitis Symptom Index.

The distribution of pain severity according to the NIH-CPSI pain score cutoffs established by Wagenlehner *et al* (21) is shown in Fig. 1A.

Similar to pain, voiding symptom scores decreased significantly at time-point V6, compared with baseline data (Table I). An additional attenuation of voiding symptoms was observed 6 months after the end of treatment (time-point V12), and was sustained through the entire follow-up, until the end of the study.

The significant improvement of voiding symptoms assessed with the NIH-CPSI was confirmed by instrumental measurement of urinary flow and voiding efficiency; mean peak flow rates increased from 14.86 ml/sec at V0 to 18.34 ml/sec at V6 and 19.02 or 18.89 ml/sec at V12 or V18, respectively (P<0.0001 for all comparisons vs. V0, or V6 vs. V12, paired, two-tailed t-test; Table I). Patients’ percentage bladder voided volume (%BVV), which was 84.53% of total bladder content at V0, was also significantly increased at V6 and V12 (98.19% and 99.61%, respectively; P<0.0001 for all comparisons vs. V0, or V6 vs. V12, paired, two-tailed t-test; Table I); this increase was sustained through the entire follow-up, until the end of the study.

Symptom relief, assessed with the NIH-CPSI test, resulted in attenuation of the impact of the disease on the QoL of patients (Table I).

The IIEF (items 1–5 plus 15) was used to assess ED in the study population. A score of 26 was used as a cutoff value to evidence mild to severe ED (15). At baseline (time-point V0) 56.9% of patients showed ED (a score <26). This proportion decreased at V6 (26.06%; P<0.0002 vs. V0, two-tailed Z test), and remained steady at subsequent time-points (V12, 26.6%; V18, 26.4%). Mean IIEF values at V0, V6, V12 and V18, and the statistical significance of intragroup comparisons, are shown in Table I.

### Subgroup analysis based on CP/CPPS sub-categories (IIIa vs. IIIb)

The study population included patients affected by either the inflammatory (IIIa) or the non-inflammatory (IIIb) sub-categories of CP/CPPS.

A comparative analysis was performed to assess any differential response to therapy between these cohorts. Table II summarizes the clinical findings of IIIa and IIIb patient cohorts at baseline, at the end of a 6-month cycle of therapy, and 6 or 12 months after the end of therapy (time-points V12 and V18, respectively). Intragroup paired analysis showed that both IIIa and IIIb patients underwent significant reductions of NIH-CPSI total scores at the end of therapy (P<0.0001 vs. V0 for both cohorts, Wilcoxon signed rank test; Table II), further improving at the V12 or V18 follow-up time-points (P<0.0001 vs. V0 and V6 for both cohorts, Wilcoxon).

Intragroup analysis of CPSI subscores showed that in both IIIa and IIIb patients pain, voiding symptoms and the impact of the disease on the QoL decreased markedly and significantly at time-points V6 (P<0.0001 vs. V0 for both IIIa and IIIb cohorts, Wilcoxon signed rank test; Table II), and decreased further at V12 or V18 (P<0.0001 vs. V0 and V6 for both cohorts, Wilcoxon).

Figure 1B shows the distribution of pain severity scores in IIIa and IIIb patients. The two groups showed a marked improvement of pain symptoms at 6 months, with no patient showing severe pain at this time-point. A small fraction of patients had moderate pain symptoms at time-point V6 (IIIa, 3.73%; IIIb, 8.78%), further decreasing at follow-up (at V12: IIIa, 1.86%; IIIb, 1.69%; at V18: IIIa, 2.98%; IIIb, 1.69%).

Uroflowmetry parameters (Qmax and %BVV) improved significantly in both groups (P<0.0001 vs. V0 for both IIIa and IIIb cohorts, paired t-test; Table II). This effect was sustained throughout the follow-up period (Table II).

Compared with IIEF average baseline values (<26 points in both IIIa and IIIb cohorts), at time-point V6 average scores were above the 26 point threshold (P<0.0001 vs. V0 for both IIIa and IIIb cohorts, Wilcoxon signed rank test; Table II). Recovery from ED was sustained throughout the follow-up period (Table II).

Intergroup analysis (CP/CPPS IIIa vs. IIIb) showed significant differences of baseline values of the NIH-CPSI total score and of pain and QoL subscores, with IIIa patients showing more severe symptoms (P<0.001 vs. IIIb for all comparisons at time-point V0, Mann-Whitney test; Table II). The ANCOVA test was used to analyze differential responses to therapy between IIIa and IIIb cohorts, as it corrects baseline imbalances and is also suitable for nonparametric analysis of data (19). As shown in Table II, analysis of symptom improvement at the end of therapy and follow-up (time-points V6 and V12/V18) evidenced significant intergroup differences. At time-point V6, pain symptoms improved more markedly in IIIa patients (mean reduction, 6.48 points), compared with IIIb patients (mean reduction, 4.69 points; P<0.0001, ANCOVA). Similarly, voiding symptoms and QoL scores improved more markedly in IIIa patients (voiding score mean reduction: IIIa, 2.5 points; IIIb, 1.63 points; impact on QoL mean reduction: IIIa, 4.32 points; IIIb, 3.06 points; P<0.0001 for both comparisons, ANCOVA). These differences concurred to generate a mean reduction of 13.23 and 9.5 points of the total NIH-CPSI score in IIIa and IIIb cohorts, respectively. Also in this case, intergroup differences were significant (P<0.0001, ANCOVA). A reduction of ≥6 points was observed at the end of therapy in 88.43% of IIIa patients and in 72.93% of IIIb patients (P<0.001, two-tailed Z-test).

Intergroup Qmax values differed at baseline, with IIIa patients showing lower peak urinary flows compared with IIIb (1.9 ml difference between IIIa and IIIb; P<0.001, unpaired t-test). At time-point V6, both groups showed a significant improvement of mean Qmax values (mean increase: IIIa, 4.15 ml/sec; IIIb, 3.01 ml/sec), but no intergroup difference was determined (P=0.48, ANCOVA). The percentage bladder voided volume, which was not different at baseline (P=0.09, unpaired t-test), increased more markedly in IIIa patients (15.69% increase in mean voided bladder volume), compared with IIIb patients (12.28% increase in mean voided bladder volume; P<0.001, ANCOVA).

Analysis of sexual dysfunction (IIEF ED scores) showed no baseline imbalances and no intergroup differences at time-point V6 (Table II). At baseline, 58.7 and 55.7% of IIIa and IIIb patients, respectively, had an IIEF ED score <26. These percentages decreased to 24.0% (IIIa) and 27.4% (IIIb) at time-point V6. Intergroup analysis showed no significant differences (P=0.6, two-tailed Z-test).

### Subgroup analyses based on differential treatment

#### Antibacterial agents

At enrollment, patients were assigned to different CP/CPPS cohorts (IIIa vs. IIIb) on the basis of the presence/absence of inflammatory findings in post-massage urine or expressed prostatic secretions. However, each study arm comprised patients subjected to different protocol treatments. All patients received α-adrenoceptor blockers and *S. repens* extracts. Patients affected by IIIb CP-CPPS not showing evidence of infection were not treated with antibacterial agents (NO-AB cohort). Patients with evidence of infection (in either the IIIa or IIIb group) were treated with antibacterial agents (AB cohort). In addition, antibacterial therapy was administered to patients with inflammatory IIIa CP/CPPS in the absence of infection, since the presence of pyuria is often suggestive of an underlying occult infection. In this respect, experts suggest empirical antibacterial therapy if infection is suspected in CP/CPPS patients (22).

Intergroup analysis limited to pre- and post-therapy data was performed to assess any differential response to combination therapy between the AB and NO-AB cohorts. Total NIH-CPSI scores decreased more markedly in the AB cohort (mean reduction, 8.51 points) compared with the NON-AB cohort (mean reduction, 4.25 points). Comparison by ANCOVA evidenced a significant intergroup difference (P=0.027). NIH-CPSI pain symptoms and the impact of the disease on QoL decreased markedly and significantly at time-point V6; however, intergroup analysis showed no significant differences between cohorts (pain, P=0.23; QoL, P=0.81, ANCOVA; data not shown).

NIH-CPSI voiding symptom scores decreased more markedly in the AB cohort (mean reduction, 2.6 points) than in the NO-AB cohort (mean reduction, 1.48 points). This intergroup difference was highly significant (P<0.0001, ANCOVA). The percentage bladder voided volume increase was significantly more pronounced in the AB cohort (mean increase, 15.83%) compared with the comparator NO-AB cohort (mean increase, 12.03%; P=0.0029, ANCOVA). Intergroup analysis of pre- and post-therapy urinary peak flow rates (AB cohort, 4.25 ml/sec increase; NO-AB cohort, 2.83 ml/sec increase) lacked statistical significance (P=0.11, ANCOVA).

Intergroup analysis of IIEF ED scores identified no significant differences between cohorts (P=0.48, ANCOVA; data not shown).

#### Phytotherapy and antioxidant supplements

CP/CPPS patients were treated with different oral preparations of *S. repens*. One preparation was based on the sole plant extract (S cohort), whereas another preparation contained the same dose of *S. repens* extract, combined with lycopene and selenium (SLS cohort). To assess any differential response to the two different *S. repens* preparations, an intergroup comparison was performed between the S and SLS cohorts. Patients treated with antibacterial agents were not included in this subgroup comparison due to their unbalanced presence in the S and SLS cohorts; analysis was limited to IIIb patients treated with α-adrenoceptor blockers and one of the two alternative *S. repens* preparations.

In the S and SLS cohorts, combination treatment induced a marked and significant improvement of CP/CPPS signs and symptoms, assessed at time-point V6 with the NIH/CPSI and IIEF questionnaires, or measured by uroflowmetry (Qmax and %BVV).

Intragroup analysis revealed significant reductions of NIH-CPSI scores in both groups (P<0.0001 for CPSI total and pain, void, and QoL subscores, Wilcoxon signed rank test), as well as highly significant improvements of the Qmax and %BVV parameters (P<0.0001 for both comparisons, paired t-test; data not shown).

Intergroup analysis, limited to pre- and post-therapy data (V0 and V6) evidenced that the arm treated with the preparation of *S. repens* combined with lycopene and selenium showed a significantly improved relief from voiding symptoms, compared with that in patients treated with *S. repens* alone. The mean improvements of voiding scores, assessed with the NIH-CPSI test, were 0.72 points for the S cohort and 1.14 points for the SLS cohort (P=0.047, ANCOVA). Average increases of Qmax at time-point V6 were 1.8 ml/sec in the S cohort and 2.6 ml/sec in the SLS cohort (P=0.019, ANCOVA). The average bladder voided volume increased by 9.2% in the S cohort and by 12.9% in the SLS cohort (P=0.011, ANCOVA).

The mean reduction of the impact of the disease on the QoL of patients, assessed with the NIH-CPSI test, was 2.21 points in the S cohort and 2.66 points in the SLS cohort. This differential response was significant (P=0.049, ANCOVA). Intergroup analysis showed that the reductions of NIH-CPSI pain and total scores were not different at the statistical level (data not shown).

## Discussion

In a prospective trial by Shoskes *et al*, it was demonstrated that 84% of patients treated with a multimodal therapy strategy addressing all six phenotypic domains of UPOINT had a reduction of ≥6 points in the total score of the NIH-CPSI symptom questionnaire (7). This important result confirmed the validity and applicability of the UPOINT algorithm for the patient-tailored diagnosis and therapy of CP/CPPS.

In the present study, a reduction of ≥6 points of the total NIH-CPSI score was achieved in 77.5% of patients subjected to combination therapy for a period of 6 months. Notably, this value is higher than the placebo effect of ~64%, demonstrated in long-term studies (23). This result shows that a clinically appreciable improvement may be achieved in a considerable fraction of patients treated with a fixed combination of agents targeting the urinary (α-adrenoceptor blockers and *S. repens*), organ-specific (*S. repens*, antioxidant/anti-inflammatory supplements and anti-inflammatory macrolides) and infection (antibacterial quinolones and macrolides, anti-biofilm and immune-modulating macrolides) domains of UPOINTS.

Acknowledging the limitations of a retrospective study design, certain comparisons and considerations may be attempted.

If an appreciable improvement can be potentially achieved in >70% of patients treated with a combination of α-blockers, antibacterial agents, plant extracts and supplements, and given that drugs targeting the psychological (antidepressants and anxiolytics), neurological (amitriptyline and pregabalin) and muscle-tenderness domains (myorelaxants) of UPOINTS can induce considerable side-effects, it could be advisable to design a simple two-step algorithm for each patient. As a first step, a combination similar to the one adopted in the present study may be administered. In instances of an unsatisfactory response (a reduction of the NIH-CPSI score <6), second-level agents such as antidepressants, anxiolytics, pregabalin, myorelaxants and others may be added to the therapeutic protocol in a second phase.

Phosphodiesterase-5 inhibitors may also be administered to patients, to address the sexual dysfunction domain of UPOINTS (8,10,12,24). Also in this case, these drugs may be administered in instances of failure of first-line agents, since the present study documents that the simple combination therapy proposed above resulted in an improvement of erectile function in approximately half (54.2%) of the patients, although phosphodiesterase-5 inhibitors were not administered during the present study. Therefore, a therapy initially targeting only the U, O and I domains of UPOINTS may in turn result in improvements of other domains, such as sexual function, in a relevant fraction of patients. This is in agreement with the evidence emerging from a previous study focusing on chronic bacterial prostatitis, a condition related to CP/CPPS, in which it was shown that combination therapy with antibacterial agents, α-blockers and *S. repens* extracts had a positive effect on the sexual function of a substantial proportion of patients (16).

Furthermore, it can be hypothesized that attenuation of the symptoms of CP/CPPS and improvement of the sexual function may have a beneficial effect on the psychological domain of UPOINTS, and in particular on disease-related anxiety and depression, potentially limiting the administration of psychoactive drugs to a smaller number of patients. Research is in progress to investigate this hypothesis.

The cohort of patients analyzed in the present study included subjects affected by the inflammatory (IIIa) and non-inflammatory (IIIb) sub-categories of CP/CPPS. This sub-classification has always been controversial, and many experts have reputed the IIIa and IIIb variants of CPPS to be equivalent if not identical conditions, based on the demonstration that the presence of leukocytes, and hence the extent of inflammation, does not correlate with the severity of symptoms of CP/CPPS, and that variable amounts of leukocytes are also retrieved in post-massage specimens of healthy/asymptomatic subjects (25,26).

In order to explore any difference in the clinical presentation and in the response to therapy of IIIa and IIIb CP/CPPS, subgroup analysis of the patient cohort was performed in the present study.

In general, patients affected by the IIIa inflammatory sub-category of CP/CPPS exhibited more severe signs and symptoms (for example, in NIH-CPSI scores and Qmax) at baseline when compared with IIIb patients. However, the improvement of symptoms was significantly more pronounced in IIIa patients than in IIIb patients. For example, a reduction of ≥6 points of the total NIH-CPSI score (14) was assessed at the end of therapy in 88.4 or 72.9% of patients affected by CP/CPPS IIIa or IIIb, respectively.

These data are not in agreement with a recent retrospective Korean study, performed on ~100 subjects, showing no baseline imbalances and no differential response to combination therapy (alfuzosin plus levofloxacin) between IIIa and IIIb CP/CPPS patients (27). Although baseline NIH-CPSI symptom scores and voiding parameters are almost identical between the Korean and the present study, comparison of the results is difficult, as in the Korean study therapy courses were shorter (6 weeks vs. 6 months in the present study), and efficacy assessments were performed at earlier time-points (6 weeks vs. 6 months in the present study).

In contrast to the Korean study and to our own previous view, the evidence emerging from the present investigation suggests that the inflammatory and non-inflammatory sub-categories of CP/CPPS may indeed represent two distinct pathological conditions or, alternatively, two different stages of the same condition. In the latter case, CP/CPPS IIIb might represent a later stage of the disease, less responsive to treatment, less prone to improvement, and characterized by a less pronounced inflammatory profile.

The differential response to therapy between IIIa and IIIb cohorts might also be due to the fact that all IIIa patients were treated with two combined antibacterial agents, whereas the vast majority (>90%) of IIIb patients did not receive antibacterial treatment. Antibacterial agents were administered to IIIb patients showing evidence of infection, and to all IIIa patients. The rationale for administering antibacterial agents to non-infected IIIa patients is based on the hypothetical presence of undetected or difficult-to-culture pathogens in prostate ducts (22).

In addition to their antibacterial activity, macrolides and, to a lesser extent, fluoroquinolones might have concurred to more marked symptom improvement in IIIa patients through their potent intrinsic anti-inflammatory properties (28,29).

Although the addition of antibacterial agents to the α-blocker/*S. repens* regime might have concurred to the improved response to therapy in IIIa patients, more severe symptoms at baseline point to a different clinical presentation of these patients, compared with subjects affected by the non-inflammatory form of the disease.

To further investigate the impact of antibacterial agents on symptom remission, the present study population was divided in two cohorts. The cohort treated with antibacterial agents included all IIIa subjects, as well as IIIb patients showing evidence of infection, whereas the remaining IIIb patients received α-blockers and *S. repens* extracts. Briefly, differential treatment resulted in different intergroup responses. The cohort treated with antibacterial agents had a more marked improvement of NIH-CPSI voiding and total scores. Voiding symptom relief was reflected by a significant improvement of peak urinary flow and bladder voiding capacity. This result supports the use of antibacterial agents in the frame of multimodal treatment of CP/CPPS, although, in contrast with published recommendations (22), our clinical group is increasingly reluctant to administer antibiotics empirically in daily practice, in the absence of documented evidence of infection.

CP/CPPS patients were treated over time with different oral preparations of *S. repens*. One kind of preparation, based on the sole plant extract, was administered in earlier years in our clinical practice. Subsequently, a preparation containing a *S. repens* extract combined with lycopene and selenium was adopted, on the basis of published evidence showing increased efficacy of this combination (17,18).

The results of the present study suggest that the addition of supplements characterized by a marked antioxidant activity may contribute to the improvement of voiding symptoms and QoL in CP/CPPS patients. These results are in agreement with the outcome of two recent randomized trials showing that a preparation of *S. repens* extracts, combined with lycopene and selenium, is more active than the plant extract alone as a symptom reliever and as a negative modulator of inflammation in CP/CPPS patients (18,19).

Within the limits of a retrospective observational study, the present results document the efficacy of the multimodal administration of diverse agents in the improvement of signs and symptoms of CP/CPPS. A reduction of ≥6 points of the NIH-CPSI score was achieved in >70% of patients, phenotyped with the novel UPOINTS system and treated with a fixed combination of α-adrenoceptor blockers, *S. repens* extracts and antioxidant supplements, to which antibacterial agents were added, in cases with evidence of prostatic infection or in the presence of inflammatory findings strongly suggestive of an ongoing occult infective process.

## Figures and Tables

**Table I:** Scores of the NIH-CPSI and IIEF symptom questionnaires, uroflowmetry data and percentage bladder voided volume in the total study population. Data are shown at enrollment (V0), at the end of a 6-month cycle of combination therapy (V6), and at follow-up 12 months (V12) and 18 months (V18) after enrollment.

	Study time-point			
	
Variable	V0	V6	V12	V18
NIH-CPSI total score [mean ± SD, (median, IQR)]	20.91±7.12 (21,10)	9.87±5.71 (9,7)^a^	8.15±4.52 (8, 4)^a^,^b^	7.62±4.13 (8, 4)^a^,^b^
NIH-CPSI pain score [mean ± SD, (median, IQR)]	9.51±3.57 (9,5)	4.08±2.67 (4,2)^a^	3.35±1.99 (3,1)^a^,^b^	3.09±1.86 (3, 2)^a^,^b^
NIH-CPSI voiding symptom score [mean ± SD, (median, IQR)]	4.01±2.59 (4,4)	2.01±1.98 (2,3)^a^	1.52±1.54 (1,2)^a^,^b^	1.45±1.53 (1,2)^a^,^b^
NIH-CPSI QoL impact score [mean ± SD, (median, IQR)]	7.40±2.81 (8,4)	3.82±2.28 (3,2)^a^	3.23±2.03 (3,2)^a^,^b^	3.02±1.77 (3,2)^a^,^b^
IIEF, items 1–5 and 15 [mean ± SD, (median, IQR)]	23.05±5.78 (24,9)	26.29±4.18 (28,4)^a^	26.06±4.92 (28,4)^a^	26.23±4.61 (28,4)^a^
Urine peak flow rate (Q_max_, ml/sec) (mean ± SD)	14.86±6.50	18.34±5.25^c^	19.02±4.20^c^,^d^	18.89±3.84^c^
Bladder voided volume (%) (mean ± SD)	84.53±18.63	98.19±7.89^c^	99.61±4.38^c^,^d^	99.57±4.03^c^

a P<0.0001 vs. V0, Wilcoxon signed rank test.

b P<0.0001 vs. V6, Wilcoxon signed rank test.

c P<0.0001 vs. V0, paired, two-tailed t-test.

d P<0.0001 vs. V6, paired, two-tailed t-test.

NIH-CPSI, National Institutes of Health Chronic Prostatitis Symptom Index; QoL, quality of life; IIEF, International Index of Erectile Function; SD, standard deviation; IQR, interquartile range.

**Table II:** Intragroup and intergroup analysis of NIH-CPSI total score and symptom domain subscores, of IIEF erectile dysfunction scores, of mean urinary peak flow levels and percentage bladder voided volumes in patients affected by the inflammatory (IIIa) and non-inflammatory (IIIb) sub-categories of CP/CPPS, assessed at time-points V0, V6, V12 and V18.

	Study time-point							
	
	V0		V6		V12		V18	
				
Variable	IIIa	IIIb	IIIa	IIIb	IIIa	IIIb	IIIa	IIIb
NIH-CPSI total score [mean ± SD, (median, IQR)]	22.22±6.99 (22,10)	19.99±7.03^e^ (20,10)	8.99±5.10 (8,5)^a^	10.49±6.03^g^ (9,8)^a^	7.86±4.39 (8,4)^a^,^b^	8.35±4.62^i^ (8,5)^a^,^b^	7.55±4.29 (7,4)^a^,^b^	7.66±4.01 (8,5)^a^,^b^
NIH-CPSI pain score [mean ± SD, (median, IQR)]	10.22±3.60 (10,5)	9.02±3.46^e^ (9,6)	3.73±2.21 (3,2)^a^	4.32±2.92^g^ (4,3)^a^	3.24±1.80 (3,2)^a^,^b^	3.43±2.12 (3,1)^a^,^b^	3.11±1.79 (3,2)^a^,^b^	3.07±1.92 (3,2)^a^,^b^
NIH-CPSI voiding symptom score [mean ± SD, (median, IQR)]	4.12±2.52 (4,4)	3.91±2.64 (4,4)	1.61±1.67 (2,2)^a^	2.28±2.13^g^ (2,4)^a^	1.38±1.52 (1,2)^a^,^b^	1.63±1.56^i^ (2,2)^a^,^b^	1.32±1.48 (1,2)^a^,^b^	1.54±1.56 (1,2)^a^,^b^
NIH-CPSI QoL impact score [mean ± SD, (median, IQR)]	7.90±2.86 (8,4)	7.05±2.73^e^ (7,4)	3.58±2.02 (3,2)^a^	3.99±2.43^g^ (4,3)^a^	3.13±1.81 (3,2)^a^,^b^	3.31±2.18^i^ (3,2)^a^,^b^	3.04±1.79 (3,2)^a^,^b^	3.01±1.76 (3,2)^a^,^b^
IIEF, items 1–5+15 [mean ± SD, (median, IQR)]	23.57±5.26 (24,8)	22.71±6.11 (24,10)	26.52±3.81 (28,3)^a^	26.15±4.44 (28,4)^a^	26.39±4.81 (28,3)^a^	25.87±5.04 (28,4)^a^	26.32±4.70 (28,3)^a^	26.02±5.66 (28,4)^a^
Urine peak flow rate (Q_max_, ml/sec) (mean ± SD)	13.76±5.30	15.60±7.11^f^	17.92±3.94^c^	18.62±5.97^c^	18.29±3.55^c^	19.55±4.55^c^,^d^	18.27±3.39^c^	19.37±4.10^c^
Bladder voided volume (%) (mean ± SD)	83.25±18.96	85.37±18.37	98.95±6.00^c^	97.65±8.97^c^,^h^	99.78±3.83^c^	99.48±4.75^c^,^d^	99.33±5.01^c^	99.76±3.10^c^

a P<0.0001 vs. V0, Wilcoxon signed rank test;

b P<0.0001 vs. V6, Wilcoxon signed rank test.

c P<0.0001 vs. V0, paired, two-tailed t-test;

d P<0.01 vs. V6, paired, two-tailed t-test.;

e P<0.001 vs. IIIa, Mann-Whitney test;

f P<0.001 vs. IIIa, unpaired, two-tailed *t*-test.

g P<0.0001 vs. IIIa, ANCOVA;

h P<0.001 vs. IIIa, ANCOVA;

i P<0.05 vs. IIIa, ANCOVA.

NIH-CPSI, National Institutes of Health Chronic Prostatitis Symptom Index; IIEF, International Index of Erectile Function; SD, standard deviation; IQR, interquartile range; QoL, quality of life; ANCOVA, analysis of covariance.
